# Emergent Cardiac Surgery With Cardiopulmonary Bypass in Early Pregnancy: Report of Four Cases

**DOI:** 10.5812/cardiovascmed.11281

**Published:** 2013-07-31

**Authors:** Bernadette Ngo Nonga, Agnès Pasquet, Laurent De Kherkove, David Glineur, Frederic Debieve, Corinne Hubinont, Gebrine El khoury, Philippe Noirhomme

**Affiliations:** 1 Service of Thoracic and Cardiovascular Surgery, Department of Cardiovascular Diseases, Brussels’s St Luc University Hospital Center, Brussels, Belgium; 2 Service of Cardiology, Department of Cardiovascular Diseases, Brussels’s St Luc University Hospital Center, Brussels, Belgium; 3 Department of Obstetrics and Gynecology, Brussels’s St Luc University Hospital Center, Brussels, Belgium

**Keywords:** Early Pregnancy, Cardiac Surgery, Cardiopulmonary Bypass, Fetal Loss

## Abstract

**Background:**

Due to current medical improvements, more women with cardiac disease are being operated during pregnancy. Fetal loss has been found to be significant between 9-30% of them and the surgery is supposed to be done maximal in the first trimester.

**Objectives:**

The aim of this study was to report our experience with urgent cardiopulmonary bypass carried out in early pregnancy and to analyze factors that may influence fetal and maternal morbidity and mortality after surgery.

**Materials and Methods:**

We have retrospectively reviewed the case notes of the patients who underwent cardiac surgery during early pregnancy in our institution from January 1997 to October 2011.

**Results:**

During that period cardiac surgery was done in 305 patients in childbearing age (between 15-50 years) from which 4 were pregnant and in the first half of their pregnancy. All of them had previous surgery due to rhumatismal heart disease .The surgery was emergent in 3 cases and urgent in 1 case. They were operated under normothermic conditions, high flow and hemodynamic stability throughout the procedure. There was no fetal loss but one patient sustained a cardiac arrest secondary to asthma complicated by post-anoxic brain injury.

**Conclusions:**

Normothermia and hemodynamic stability are the most important factors which help to reduce fetal loss during open heart surgery in pregnancy. The fetus has an auto-regulation which comes into play when the mother is experiencing shock.

## 1. Background

As life expectancy increased and medical management improves there are more women in childbearing age diagnosed with a cardiac disease (1). In Western countries, congenital heart disease have been reported to be one of the most common causes of cardiac pathology during pregnancy, but rheumatismal heart disease is not uncommon due to immigration (2). Some of these patients may develop life threatening complications like valve thrombosis, acute aortic or coronary dissection or cardiac failure for which an urgent or emergent operation is necessary to save the life of the mother and the fetus (3, 4).With current medical practice, the risk of cardiac surgery during pregnancy for the mother is the same as to the non-pregnant women except in emergencies where cardiac surgery during pregnancy carries a higher morbidity and mortality than in non-parturient patients (3). The reported fetal loss is 9-30% (1, 3, 4) and the risk was found to be higher for the fetus when the surgery is done earlier in the pregnancy (4). Hemodynamic changes during pregnancy put a burden on the heart with cardiac diseases being responsible of more than 50% of the non-obstetrical mortality during pregnancy (1, 2, 4). Some physicians still recommend abortion when a patient with cardiac disease gets pregnant (5), although the European Society of Cardiology (ESC) recommends cardiac surgery during pregnancy when medical management fails (6). The best time for cardiac surgery during pregnancy has been reported to be between 24-28 weeks (4).

## 2. Objectives

In this study we are reporting our experience in the management and the progression of pregnancy in patients who underwent urgent cardiopulmonary bypass in the first half of their pregnancy.

## 3. Patients and Methods

We have reviewed the medical records of all the patients who underwent cardiac surgery during the first half of the pregnancy in our cardiovascular surgical service of the university hospital St Luc (Catholic University of Leuven) in Brussels from January 1997 to October 2011. The charts were reviewed for demographic data, cardiac pathology, surgical procedure, postoperative complications and pregnancy outcome. The initiation of bypass was done after full heparinisation (3mg/kg) and an ACT of 450 was necessary for the bypass; we used blood anterograde cardioplegia in all procedures. Initial pump prime additive were crystalloid and artificial colloid. Normothermic flow was maintained throughout the procedure, and care was taken to avoid hypotension. The flow was maintained above 2, 5 L/m^2^/min and Increasing flow rate with crystalloid was used in case of hypotension. All efforts were made to maintain hemodynamic stability throughout the procedure. No fetal monitoring was undertaken because this is best done after the 18^th^ week of pregnancy. We collected additional data from follow up consultation and by phone calls to patients; informed consents were obtained from the patients contacted and the study has received approval of the hospital’s internal review board.

## 4. Results

From January 1997 to October 2011, 305 women in childbearing age (15 to 50 years) underwent cardiac surgery from which 4 were in their first half of pregnancy during the cardiopulmonary bypass (CPB) with an incidence of 1, 64%. The mean age was 30.25 (22-37) years. The median gestational age (GA) was 11.5 (8-15) weeks. The cardiac pathology was acquired in all patients and it was secondary to rheumatic fever. A 22 years old young woman with mitral and aortic valve rheumatismal disease was proposed abortion by her cardiologist after she became pregnant. Since she wanted to keep her pregnancy she was referred to our service for a second opinion and her case was discussed during a multidisciplinary staff where a decision was made to do the multi valve replacement as she was already in the class III of New York Heart Association (NYHA). The surgery was done urgently when she was 8 weeks pregnant. All four patients have a history of previous cardiac surgery: mitral or aortic valve replacement (Table 1).

**Table 1.** Past Medical, Obstetrical and Cardiac Histories

Age	Past	Medical and Obstetrical History	NHYA	Gestational Age
**30**	CVA-Stroke in 1997, three childrenborn in (1998, 2000, 2002), HTA	AVR^a^, Mec^a^1994	Class I	9 weeks
**36**	Two previous children:Baby girl bornin 1998,38 w 2, 9 kg. Baby boy born in 2006, 36w, 2800g, Asthma, cardiacarrest	MVR^a^2005, mec	Class IV	15 weeks
**33**	Miscarriage at 6 weeks,Atrial fibrillation	MVR 2005, mec	Class I	14 weeks
**22**	none	MVR, Bio^a^2006	Class III	8 weeks

^a^Abbreviations: AVR, aortic valve replacement; MVR, mitral valve replacement; Bio, bioprosthesis; Mec, mechanical

They underwent five procedures: one patient was taken back to the operating room because she developed a severe acute aortic insufficiency with cardiac failure 2 days after a mitral valve replacement. The indication for surgery was a mitral mechanical valve thrombosis in 2 patients, an aortic mechanical valve thrombosis in one patient and a biological mitral valve stenosis and native aortic valve stenosis in one patient with rheumatismal heart disease. The mechanical valve thrombosis occurred while patients were placed on low molecular weight heparin, despite a closed follow up and an optimal anti Xa activity. Maternal history prior to the surgery and the NYHA classification are displayed in Table 2.

**Table 2.** Type of Pathology, Procedure and Duration of Bypass

Age	G A^a^	Current Pathology	Date of Surgery	Procedure	Duration of Bypass/C-C^a^
**30F**	9w	AV^a^ thrombosis	18/12/1997	Ross procedure^a^	NA
**36F**	15 W	MV^a^ thrombosis	24/11/2010	MVR^a^Bio^a^	188/88
**36F**	15 W	AI^a^	26/11/2010	AVR^a^	63/39
**33 F **	14 W	Thrombosis of both valves	3/3/2011	MVR , AVR, Bio	91/71
**22F**	8 W	AV stenosis, MV stenosis	6/10/2011	Ross and Mitral Valvuloplasty	170/156

^a^Abbreviations: AI; Aortic Insufficiency ,AV; Aortic Valve, AVR, aortic valve replacement; MV; Mitral Valve , MVR, mitral valve replacement; Ross Procedure, aortic pulmonic valve autograft associated with pulmonic homograft; Bio, bioprothesis; GA, gestational age; Duration of bypass /C-C, crossclamping in minutes.

The procedures were done in an urgent and emergent basis. The surgery on thrombosed valves were emergent procedures and the mitral biological valve and aortic stenosis replacement were done on an urgent basis. The surgery was undertaken under general anesthesia using the regular drug for cardiac surgery. All patients had a history of previous sternotomy and one patient was operated three times in the past. Arterial and venous cannulation was done using the aorta and both vena cavas except in the patient with the multiple previous sternotomies, where the cannulation was done using the femoral vessels. The mean duration of the bypass was 128 (63-188) minutes and the cross-clamping time was 88, 5 (39-156) minutes (Table 2). The mean hemoglobin preoperatively was 10.8(8.7-12.2) g/l, the lowest hemoglobin during CPB was 7.5 (6.5-9) g/l. The hemoglobin at the end of the case was 8.6 (8.2-9.5) g/l. Only one patient was transfused with 2 fresh frozen plasma and 2 packs of red blood cells. 

Maternal outcome: the surgical procedure and post-operative course were uneventful in three patients with very good results, and patients resuming the NYHA Classification I. A 36 years old asthmatic woman admitted for cardiac failure secondary to a thrombosed mechanical mitral valve who was on low molecular weight heparin (LMWH) with a close follow-up had a mitral valve replacement using a bioprothesis. After a very difficult procedure due to previous operation, she remained hemodynamically unstable in the immediate post-operative period and amine vasopressor dependent due to severe aortic insufficiency. She was taken back to the operating room two days after and underwent an aortic valve replacement using a bioprothesis valve. She was extubated 2 days after the surgery. Three days later she had a cardiac arrest secondary to a severe bronchospasm exacerbation by bilateral vocal cord palsy secondary to the intubation. She remained in a comatose state and in the intensive care unit for few months. The MRI showed evidence of post anoxic cerebral lesions. While in the intensive care unit, she developed multiple hospital acquired lung and urinary tract infections due to staphylococcus, pseudomonas, candidiasis and klebsiella pneumonia treated with vancomycin, Meropenem, Fluconazole, ciprofloxacine and ticarcillin. She remained in a vegetative state until the 32 ^th ^week of pregnancy when she developed premature labor with uterine contractions because of a placenta preavia. These were calmed by the administration of Beta adrenergic medications and she received steroids for lung maturation. She underwent a cesarian- section (C-section) on the 34 ^th ^week. The baby was a boy, , apgar 7 at 5min and 8 at 10 min; he was admitted in the neonatology unit. The patient was discharged to a neurological center 2 weeks after the delivery in the same vegetative state and she is still alive at present. Fetal outcome: The follow up was from 12 months to 14 years (Table 3).

**Table 3.** Clinical Presentation, Fetal and Maternal Outcome

Age	Gestational Age	NHYA	Fetal Outcome	Maternal Outcome	Length of Hospital Stay
**30**	9 weeks	Stage I	Vaginal delivery, baby girl 2,8kg born 38weeks, under LMWH, no complications Alive and well	No complication	unknown
**36**	15 weeks	Stage IV	Placenta praevia, premature labor, C-SECTION at 34 W, baby girl, 1600g, apgar7 and 8.Fetal MRI before C section was Alive and well	Post anoxic brain injury	4 months
**33**	14 weeks	Stage I	C-section delivery 38 W for arrested labor, baby boy weight 398Og, Apgar 10 and 10 Alive and well	No complication	7 days
**22**	8 weeks	Stage III	Normal Pregnancy , vaginal delivery at 40 weeks, normal baby girl 3,3kg, apgar 10/10	No complication	12 days

Three women delivered healthy babies: two by vaginal delivery at 38 weeks and 40 weeks, and the other one by C-section: There was one premature labor where the patient underwent C-section at 34 weeks after uterine contractions started at 32 weeks. All these children are still alive and doing well. The pathological analysis of the placenta was normal, it did not show any significant abnormality.

## 5. Discussion

In this study, women in the first half of the pregnancy represented 1.6% of all women of childbearing age undergoing cardiac surgery in our center. The mean age of mothers was the same as the one reported by many series (3). In western countries, many of these patients have congenital heart diseases, although rheumatismal heart disease is not uncommon due to immigration as in the present study where all our patients presented complicated rhumatismal heart diseases.

Jacob et al first reported cardiac surgery in the first trimester of pregnancy in three cases: survival of the mother and the fetus in 1965 was 100% in the postoperative period up to delivery, one mother died of massive cardiac failure 2 days after delivering a healthy baby (7). Maternal death have been found to be minimal since 1982 (8) ,In that study, Becker et al. have found that the fetal mortality was highest with valve replacement and they have recommended using high , normothermic flow to reduce complications in the fetus. With current days practices as in this study the survival of the fetus is even higher (100% in this study).

Multiple recognized risk factors for complications in the mother and the fetus were found in all our patients: reoperation, emergent surgery, left heart procedure and simultaneous aortic and mitral valve replacement (3, 4). Despite all these risk factors, only one patient was taken back to the operating room and subsequently sustained a cardiac arrest with development of post anoxic brain injury. But this patient had multiple co-morbidities, she was above 35 years of age and she underwent CPB 2 times in 48 hours. The cardiac arrest was secondary to severe bronchospasm and asthma that was present in her past medical history. The fetus sustained the hemodynamic instability and the cardiac arrest in the mother, making us hypothesizing that it has an intrinsic auto regulation and may have adaptive measures in face of intense stress in the mother mediated by the hypothalamus (9, 10). Pregnancy loss during cardiac surgery must be due to many factors and may be the result of an imbalance between the duration of the stress and the fetus auto-regulation. Alcazar et al have reported the case of a mechanical prosthetic mitral valve thrombosed during early pregnancy who underwent a successful valve repair using cardiopulmonary bypass and mild hypothermia (11).

Fetal loss have been reported to be variable from 9 to 30% (3, 4) and 24% under hypothermic conditions and this loss after cardiac surgery has been reported to be highest during the first trimester (4) Renato at al. have found that fetal death is related to maternal age, emergent operation, reoperation and NYHA functional class (12), others have stressed that younger gestational age, hypotension and hypothermia, are the most important risk factors. Although hypotension and hypothermia may be deleterious to the fetus, at later gestational ages, there are other factors which must come into play when there is a strong stress in the mother, early during the pregnancy. There is no malformation or congenital abnormality that we can report in the fetus making it difficult for us to study the possible teratogenic effect of the CPB. Maybe the fetus in our cases did well because the mothers have rheumatismal heart disease rather than congenital heart disease (13).

The duration of the bypass and cross-clamping time were longer than usually reported (3), because our patients underwent multiple valve surgery and redo surgery, three of the four patients had double valve surgery. We used well standard precautions to decrease the complications rate in our patients: normothermia, avoiding hypotension and ensuring a good hemoglobin level for optimal oxygen carrying capacity, as previous authors have found (3, 4, 13). Our data supports the fact that fetal safety is the highest when using normothermia and high flow circulation.

Although the management was optimal by using the safest recommendations: normothermia, normal mean perfusion pressure and keeping an optimal oxygen carrying capacity of the blood, the strength of the management relies on a multidisciplinary team approach of all the people involved in taking care of a pregnant woman with a surgical cardiac condition: surgeons, cardiologist, obstetricians, anesthesiologist, intensive care physicians, psychologists and more. Those patients were followed up closely from the time of the diagnosis to the completion of the pregnancy. We still recommend aggressive medical treatment for patients with cardiac diseases during pregnancy, surgery being reserved for intractable cases. But surgery should not be postponed or abortion proposed when this treatment failed, because in experienced centers this surgery can be performed with safety for both the mother and the fetus. This study is a retrospective study with a small number of patients and as such it carries some limitations. However this study highlights the facts that fetal loss during early pregnancy can be decreased using optimal operative conditions which include normothermia, normal perfusion pressure and an optimal oxygen carrying capacity in the blood and the fact that fetus has an intrinsic autoregulation in case of intense stress in the mother.
